# CD26^low^PD-1^+^ CD8 T cells are terminally exhausted and associated with leukemia progression in acute myeloid leukemia

**DOI:** 10.3389/fimmu.2023.1169144

**Published:** 2023-06-29

**Authors:** Huarong Zhou, Bei Jia, Charyguly Annageldiyev, Kentaro Minagawa, Chenchen Zhao, Shin Mineishi, W Christopher Ehmann, Seema G. Naik, Joseph Cioccio, Baldeep Wirk, Natthapol Songdej, Kevin L. Rakszawski, Myles S. Nickolich, Jianzhen Shen, Hong Zheng

**Affiliations:** ^1^ Penn State Cancer Institute, Penn State University College of Medicine, Hershey, PA, United States; ^2^ Fujian Institute of Hematology, Fujian Provincial Key Laboratory of Hematology, Fujian Medical University Union Hospital, Fujian Medical Center of Hematology, Fuzhou, China; ^3^ Department of Microbiology and Immunology, Penn State University College of Medicine, Hershey, PA, United States

**Keywords:** AML, PD-1, CD26, t cell exhaustion, terminal exhaustion

## Abstract

Acute myeloid leukemia (AML) is a devastating blood cancer with poor prognosis. Novel effective treatment is an urgent unmet need. Immunotherapy targeting T cell exhaustion by blocking inhibitory pathways, such as PD-1, is promising in cancer treatment. However, results from clinical studies applying PD-1 blockade to AML patients are largely disappointing. AML is highly heterogeneous. Identification of additional immune regulatory pathways and defining predictive biomarkers for treatment response are crucial to optimize the strategy. CD26 is a marker of T cell activation and involved in multiple immune processes. Here, we performed comprehensive phenotypic and functional analyses on the blood samples collected from AML patients and discovered that CD26^low^PD-1^+^ CD8 T cells were associated with AML progression. Specifically, the percentage of this cell fraction was significantly higher in patients with newly diagnosed AML compared to that in patients achieved completed remission or healthy controls. Our subsequent studies on CD26^low^PD-1^+^ CD8 T cells from AML patients at initial diagnosis demonstrated that this cell population highly expressed inhibitory receptors and displayed impaired cytokine production, indicating an exhaustion status. Importantly, CD26^low^PD-1^+^ CD8 T cells carried features of terminal exhaustion, manifested by higher frequency of T_EMRA_ differentiation, increased expression of transcription factors that are observed in terminally exhausted T cells, and high level of intracellular expression of granzyme B and perforin. Our findings suggest a prognostic and predictive value of CD26 in AML, providing pivotal information to optimize the immunotherapy for this devastating cancer.

## Introduction

Acute myeloid leukemia (AML) is a devastating blood cancer with poor prognosis. Although treatment of AML has been significantly advanced recently with several novel targeting agents approved by FDA, five-year overall survival remains low at only 30.5% (1). Novel effective treatment is clearly an unmet need.

Multiple studies including ours have demonstrated the involvement of T cell exhaustion in AML pathogenesis (2–9). A recent study showed that T cell exhaustion may be a predominant process in AML at diagnosis and AML shaped CD8 T cell response *in vitro (* 10). Up-regulation of PD-1 and other immune inhibitory pathways, the hallmark for T cell exhaustion, was found to be associated with AML progression (5). Importantly, PD-1 blockade enhanced T cell activity and reduced leukemia burden in mouse models of AML (6, 9). These observations suggest an important role of T cell exhaustion in AML. However, clinical studies applying PD-1 blockade to AML patients showed limited benefit (11–13). AML is highly heterogeneous. Identification of additional immune regulatory pathways and defining predictive biomarkers for treatment response are crucial to optimize treatment targeting T cell exhaustion and develop effective immunotherapy for AML.

CD26, also known as dipeptidyl peptidase 4 (DPP4), is a homodimeric type II transmembrane glycoprotein expressed on many cell types, including epithelial cells and immune components such as T cells, B cells, NK, and macrophages (14–18). CD26 is multifunctional and is involved in glucose homeostasis (19), stem cell homing (20), regulation of inflammatory diseases and multiple immune processes (21). CD26 is a marker for T cell activation. It acts as a costimulatory molecule enhancing interactions between antigen-presenting cells and T cells, subsequently initiating the signal transduction process and promoting T cell activation. Up-regulation of CD26 has been observed on both CD4 and CD8 T cells that are highly function in antiviral and anti-tumor response (22). However, the impact of T cell expression of CD26 on AML has not been studied. To fill this gap, we examined T cells of peripheral blood collected from a cohort of newly diagnosed AML patients (n=28). Subpopulations of T cells expressing different level of CD26 were further dissected for their phenotypic and functional status, as well as correlations with clinical outcome.

## Materials and methods

### Patient

Peripheral blood and bone marrow samples were collected from AML patients diagnosed per WHO criteria. All the patients were diagnosed at the Penn State Hershey Cancer Institute of Penn State University College of Medicine (Hershey, PA, USA). The study was approved by the Institutional Review Board of Penn State College of Medicine. Fully informed consent was obtained from all patients.

### Isolation of PBMCs

Peripheral blood and bone marrow samples were collected from patients with newly diagnosed AML (n=28), AML patients in complete remission (n=15), and healthy donors (n=18). Peripheral blood mononuclear cells (PBMCs) and bone marrow mononuclear cells were isolated by density gradient centrifugation using Ficoll-Paque (Amersham Pharmacia Biotech, Stockholm, Sweden). Cells were preserved in fetal bovine serum containing 10% dimethyl sulfoxide (Gibco, Grand Island, NY, USA) and stored in liquid nitrogen.

### Immunofluorescence staining and flow cytometry analysis

For surface staining, frozen PBMCs were thawed at 37°C and washed 2 times with phosphate-buffered saline containing 1% fetal bovine serum. Cells were incubated with Human BD Fc Block™ (10 minutes at room temperature) followed by staining with directly conjugated mAbs for 30 minutes at 4°C. Cells were then washed and resuspended in staining buffer before flow cytometry analysis. The monoclonal antibodies used were anti-human CD3-BV605, CD4-BV711, CD8-APC-H7, CD45RA-AF700, CD26-PE-CF594 or CD26-BV421, Ki67-AF488, Granzyme B-AF700, T-bet-PE, TCF-7/TCF-1-AF647, CD95-BV421, Annexin V-PE, hCD45-BV605 (BD Biosciences, San Diego, CA, USA), CCR7-BV421, PD-1-BV785, CD226-FITC, TIM-3-PE-Cy7, Perforin-APC (Biologend, San Diego, CA, USA), TIGIT-APC, Eomes-PE-eF610, TOX-PE, AITR/GITR-PE (invitrogen, Carlsbad, CA, USA) antibodies and corresponding isotype controls. Data were acquired using an LSR Fortessa flow cytometer (BD Biosciences) and analyzed with FlowJo software (Tree Star, Ashland, OR, USA).

### *In vitro* stimulation and intracellular cytokine staining

PBMCs were cultured in RPMI-1640 medium (Gibco) containing 10% fetal bovine serum and stimulated with anti-CD3/CD28 (2 and 2.5 μg/mL) at the presence of Plus Golgiplug (BD Pharmingen, San Diego, CA, USA) for 5 hours. Cell viability was assessed using the Fixable viability dye eFluor^TM^ 506 (invitrogen, Carlsbad, CA, USA). Cells were then surface stained with CD4-FITC, CD8-APC-H7, PD-1-BV785, and CD26-PE-CF594. After fixation and permeabilization, intracellular staining was performed with IL-2-PE-Cy7, TNF-α-BV421, IFN-γ-APC (BD Biosciences) antibodies.

### Statistical analysis

All summary statistics (average values, s.d., s.e.m., significant differences between groups) were calculated using GraphPad Prism 9 (GraphPad Software Inc., San Diego, CA) or SPSS Statistics 26 as appropriate. For data distributed normally, the comparison of variables was performed using unpaired or paired Student t test. For data not distributed normally, the comparison of variables was performed with a Mann–Whitney U test or a Wilcoxon signedrank test for unpaired and paired data, respectively. Comparisons of categorical patient characteristics were analyzed using Fisher exact test. The ROC curve was used to predict the reasonable grouping cutoff of low CD26^low^PD-1^+^ and high CD26^low^PD-1^+^ in newly diagnosed AML patients and healthy controls. The overall survival was analyzed by the log-rank (Mantel–Cox) test. For all analyses, a P value of < 0.05 was considered statistically significant.

## Results

### The proportion of CD26^low^PD-1^+^ CD8 T cells is significantly higher in blood of patients with untreated AML

**Figure 1:**
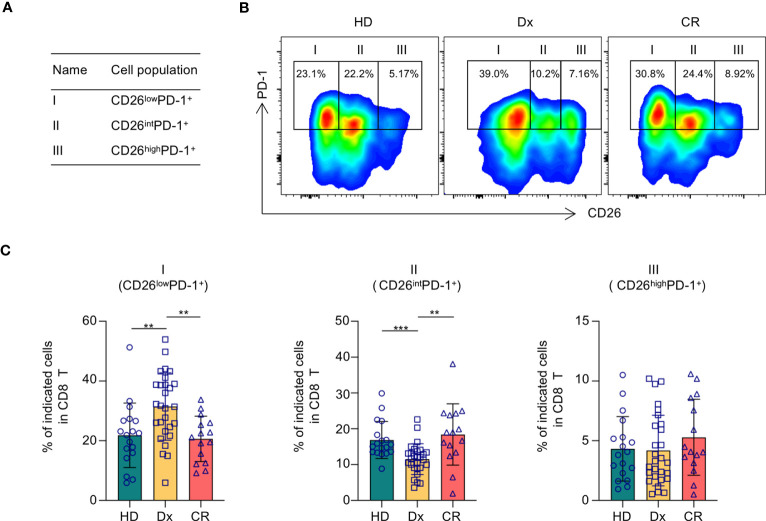
CD26^low^PD-1^+^ CD8 T cells are significantly increased in AML patients at newly diagnosis. PBMCs collected from healthy donors, AML patients at newly diagnosis and in complete remission were assessed for CD26 and PD-1 expression on CD8 T cells by flow cytometry. **(A)** Based on the levels of CD26 and PD-1 expression, CD8 T cells are divided into three fractions. Shown is the schema of each fraction. **(B)** Representative flow data from healthy donors (HD), AML patient at newly diagnosis (Dx) and in complete remission (CR) displaying the percentage of CD26^low^PD-1^+^(fraction I), CD26^int^PD-1^+^ (fraction II), CD26^high^PD-1^+^ (fraction III) among CD8 T cells. **(C)** The frequencies of each fraction among CD8 cells in HD(n=18), AML patients at initial diagnosis (Dx, n=28) and AML patients in complete remission (CR, n=15). Each spot represents data of an individual patient or healthy donor. *P* values were obtained by unpaired Student t-test or Mann-Whitney test. **P<0.01, ***P<0.001.

Given its costimulatory function in T cell activation, we initially hypothesized that down-regulation of CD26 on T cells correlates with T cell hypofunction and subsequently AML progression. We performed flow cytometry analyses on PBMCs collected from AML patients at newly diagnosis (n=28) vs. that of healthy controls (n=18). The clinical characteristics of the AML patients are summarized in **Table 1**. Consistent with the heterogeneity of AML, there was wide variability in white blood cell (WBC) counts and blast percentages in the peripheral blood. The majority of patients carried intermediate or adverse cytogenetic features. Surprisingly, no significant differences in T cell expression of CD26 were observed (**Supplemental Figure 1**). However, when PD-1 was added to the analyses, in which PD-1^+^ T cells were divided into 3 subsets based on the expression of CD26 (**Figure 1A**), we made striking observation that the frequency of PD-1^+^ CD8 T cells expressing low level of CD26 (CD26^low^PD-1^+^) were significantly higher in newly diagnosed AML patients compared to that in healthy controls (31.45 ± 2.129% vs. 21.83 ± 2.541%, *P*=0.0062; **Figures 1B, C**). We further examined PBMCs from AML patients who have achieved complete remission (CR) after chemotherapy (n=15), and found that similar to healthy controls, CD26^low^PD-1^+^ CD8 T cells in these patients are significantly lower than that in newly diagnosed AML. In contrast, the frequency of CD8 T cells expressing intermediate level of CD26 (CD26^int^PD-1^+^) were lower in newly diagnosed AML compared to AML in CR or healthy controls (**Figures 1B, C**). Same analyses were performed on CD4 T cells and no significant differences were observed (**Supplemental Figures 1, 2**). These data suggest that CD26^low^PD-1^+^ CD8 T cells correlate with AML progression.

**Table 1:** Clinical feature of the AML patients.

Variable	Value
Age, y	
Median	63
Range	23-79
Gender, n (%)	
Male	10(36)
Female	18(64)
WBC, ×10^9/L	
Median	54.45
Range	3.6-361
PB blasts (%)	
Median	65
Range	12-94
Absolute blasts count, ×10^9/L	
Median	27.59
Range	0.71-315.88
Cytogenetics*, n (%)	
Favorable	2(7)
Intermediate	16(57)
Adverse	10(36)

WBC, white blood cell; PB, peripheral blood.

*Risk stratification is per ‘2022 ELN risk classification by genetics at initial diagnosis (23).

### Terminally differentiated effector cells are significantly increased in CD26^low^PD-1^+^ CD8 T cells

**Figure 2:**
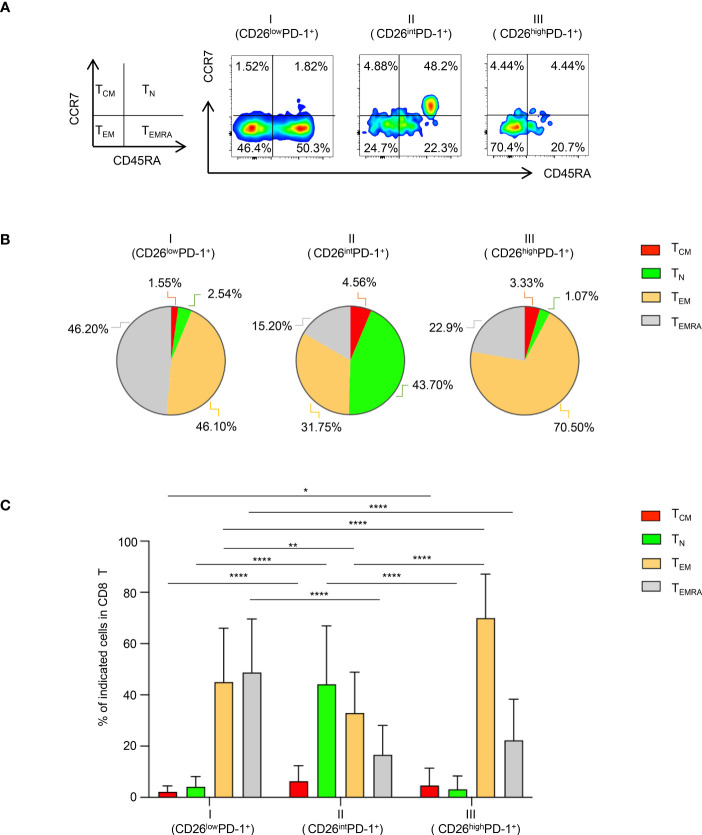
Terminally differentiated effector cells are significantly increased in CD26^low^PD-1^+^ CD8 T cells. Flow cytometry analysis of surface expression of PD-1, CD26, CD45RA, CCR7 was performed on PBMCs collected from AML patients at initial diagnosis. **(A)** The gating strategy of Naïve (T_N_), central memory (T_CM_), effector memory (T_EM_) and terminal differentiated cells (T_EMRA_) in CD8 T cells was shown on the left. The representative flow images on the right show the distribution of the above subsets in CD26^low^PD-1^+^ (fraction I), CD26^int^PD-1^+^ (fraction II) and CD26^high^PD-1^+^ (fraction III) in CD8 T **(B)** The pie chart depicts the distribution of T_N_, T_CM_, T_EM_ and T_EMRA_ in CD26^low^PD-1^+^ (fraction I), CD26^int^PD-1^+^ (fraction II) and CD26^high^PD-1^+^ (fraction III) CD8 T cells. **(C)** Summary data for the distribution of naïve vs. memory in the three fractions of CD8 T cells. *P* values were obtained by paired Student t-test or Wilcoxon signed rank test. *P<0.05, **P<0.01, ****P<0.0001.

We then focused our study on characteristic analyses of CD26^low^PD-1^+^ CD8 T cells. PBMCs from patients with newly diagnosed AML were examined. We first assessed the differentiation status of this cell population. Based on the expression of CD45RA and CCR7, T cells can be divided into four differentiation subsets (**Figure 2A**): naïve T cells (T_N_, CCR7^+^CD45RA^+^), central memory T cells (T_CM_, CCR7^+^CD45RA^−^), effector Memory T cells (T_EM_, CCR7^−^CD45RA^−^) and terminally differentiated effector cells (T_EMRA_, CCR7^−^CD45RA^+^). We performed multichannel flow cytometry analyses to dissect the distribution of all four differentiated subsets in CD26^low^PD-1^+^ CD8 T cells as well as the other two PD-1^+^ CD8 T cell populations based on CD26 expression (CD26^int^PD-1^+^ and CD26^high^PD-1^+^). Consistent with the previous report that most CD26^int^ T cells are naïve, we found a high frequency of naïve cells in CD26^int^PD-1^+^ CD8 T cells from our AML patients. In contrast, both CD26^low^PD-1^+^ and CD26^high^PD-1^+^ CD8 T cells are antigen experienced. Importantly, we observed a significantly higher frequency of T_EMRA_ cells in CD26^low^PD-1^+^ than CD26^high^PD-1^+^ CD8 T cells (48.74% vs. 22.28%, *P*<0.0001; **Figures 2B, C**). T_EMRA_ is considered to be a terminal effector cells with limited function. This data suggests an association of CD26^low^PD-1^+^ CD8 T cells with T cell dysfunction and AML pathogenesis.

### Expression of inhibitory receptors is increased on CD26^low^PD-1^+^ CD8 T cells

**Figure 3:**
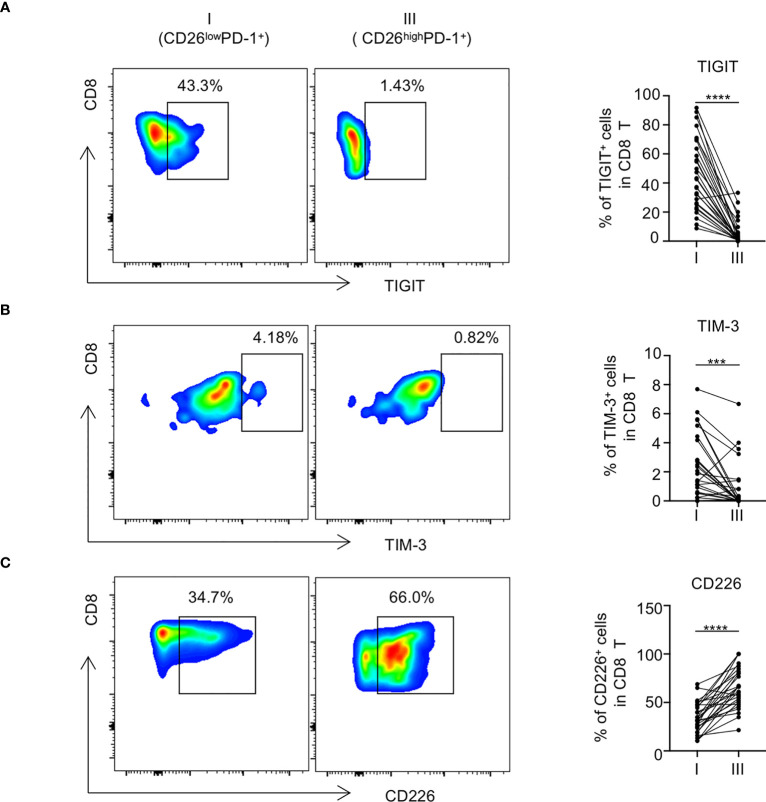
The expression of inhibitory receptors in CD26^low^PD-1^+^ CD8 T cells in newly diagnosed AML patients was increased. PBMCs from AML patients at initial diagnosis were examined by flow cytometry(n=28). The expression of TIGIT [shown in **(A)**], TIM-3 [shown in **(B)**] and CD226 [shown in **(C)**] on CD8 T cell subpopulations [CD26^low^PD-1^+^(fraction I) and CD26^high^PD-1^+^ (fraction III)] was assessed. **(A–C)** Shown are the representative flow data (left) and summary plots (right). P values were obtained by paired Student t-test or Wilcoxon signed rank test. ***P<0.001, ****P<0.0001.

We next examined the impact of CD26 expression on the inhibitory and stimulatory pathways in PD-1^+^ CD8 T cells. To rule out potential confounding effect of Naïve T cells, we excluded Naïve-dominant CD26^int^PD-1^+^ CD8 T cells and focused our subsequent analyses on antigen experienced cells including CD26^low^PD-1^+^ and CD26^high^PD-1^+^ CD8 T cells. When surface expression of a number of inhibitory receptors on these two cell populations was compared, we observed significantly higher expression of TIGIT and TIM-3 on CD26^low^PD-1^+^ CD8 T cells, compared with that of CD26^high^PD-1^+^ CD8 T cells (TIGIT: 46.09% vs. 5.71%, *P*<0.0001; TIM-3: 2.28% vs. 0.34%, *P*=0.0001; **Figures 3A, B**). In contrast, expression of the stimulatory receptor CD226 (counterpart of TIGIT) was significantly lower on CD26^low^PD-1^+^ CD8 T cells (34.4% vs. 65.3%, *P*<0.0001, **Figure 3C**). Up-regulation of inhibitory receptors is a hallmark of T cell exhaustion. Our finding indicates that CD26^low^PD-1^+^ CD8 T cells are likely in a more advanced exhaustive status, compared with CD26^high^PD-1^+^ CD8 T cells.

### CD26^low^PD-1^+^ CD8 T cells express higher level of exhaustion related transcription factors

**Figure 4:**
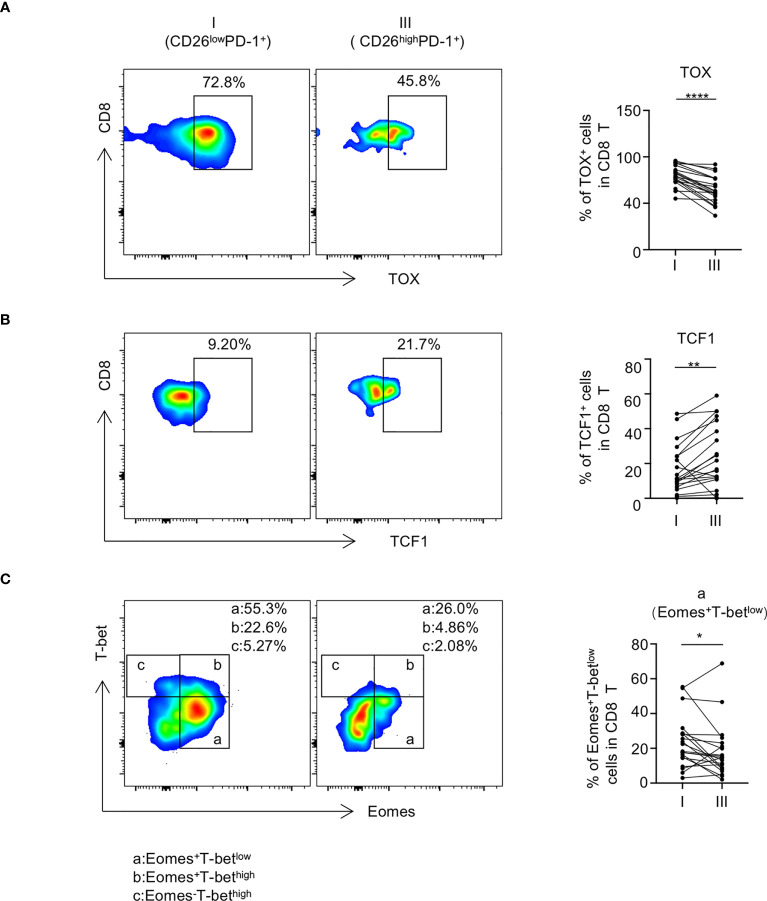
CD26^low^PD-1^+^ CD8 T cells express higher level of exhaustion-related transcription factors. PBMCs collected from AML patients at initial diagnosis were assessed by flow cytometry (n=20). The expression of TOX [shown in **(A)**], TCF1 [shown in **(B)**] Eomes and T-bet [shown in **(C)**] on CD8 T cell subpopulations [CD26^low^PD-1^+^ (fraction I) and CD26^high^PD-1^+^ (fraction III)] was analyzed by flow cytometry. Upon the expression of Eomes and T-bet, CD8 T cells were divided into subsets of Eomes^+^T-bet^low^(a), Eomes^+^T-bet^high^ (b) and Eomes^-^T-bet^high^ (c), **(A–C)** Flow cytometry representative data were shown on the left and summary plots were shown on the right. *P* values were obtained by paired Student t-test or Wilcoxon signed rank test. *P<0.05, **P<0.01, ****P<0.0001.

We further assessed the expression pattern of transcription factors in CD26^low^PD-1^+^ CD8 T cells. Studies in models of chronic viral infection have demonstrated that several transcription factors including Eomes, T-bet, TOX, and TCF1 are important in regulating T cell exhaustion. We compared the intracellular expression of these transcription factors in CD26^low^PD-1^+^ CD8 T cells to that in CD26^high^PD-1^+^ CD8 T cells. As shown in **Figure 4A**, we observed a higher percentage of TOX expression in CD26^low^PD-1^+^ CD8 T cells (79.77% vs. 64.55%, *P*<0.0001). In contrast, expression of TCF1 in CD26^low^PD-1^+^ CD8 T cells was significantly lower (16.73% vs. 24.06%, *P*=0.0023, **Figure 4B**). When expression of Eomes and T-bet was assessed, we focused our analyses on the Eomes^+^T-bet^low^ subset as our previous work showed that this subset was associated with poor clinical outcome in AML patients (4). As shown in **Figure 4C**, we observed a higher percentage of Eomes^+^T-bet^low^ cells in CD26^low^PD-1^+^ CD8 T cells, compared to that in CD26^high^PD-1^+^ CD8 T cells (23.64% vs. 18.18%, *P*=0.0233). Collectively, we found that CD26^low^PD-1^+^ CD8 T cells from untreated AML patients express higher level of TOX and Eomes, whereas lower level of TCF1. This transcription pattern is more consistent with terminal exhaustion.

### CD26^low^PD-1^+^ CD8 T cells exhibit functional defects

**Figure 5:**
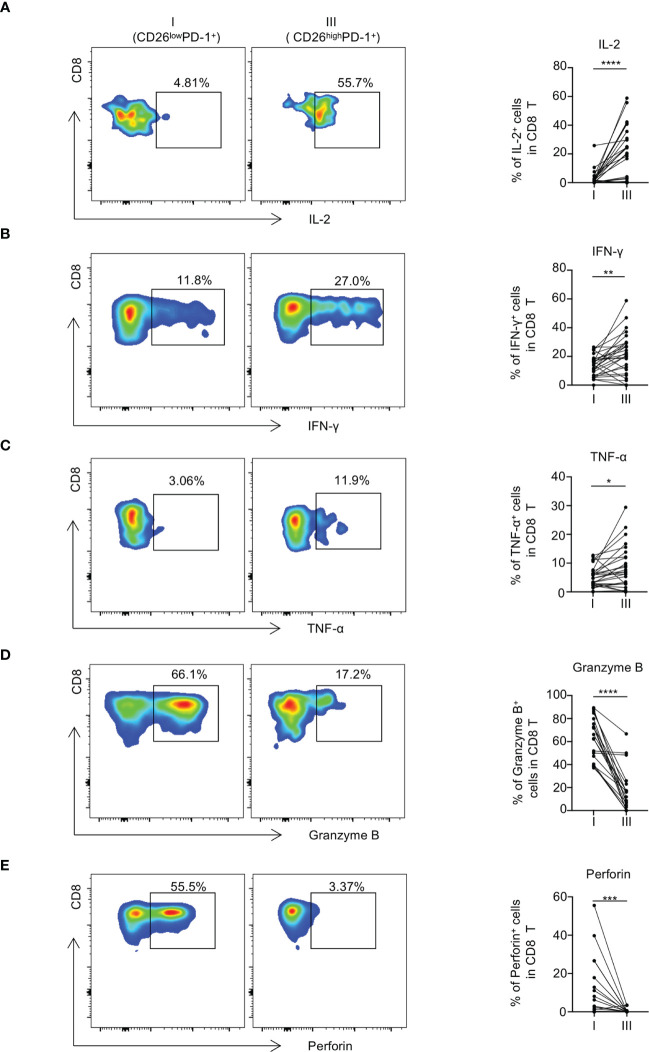
CD26^low^PD-1^+^ CD8 T cells produce less cytokines and display decreased cytotoxic capacity in AML patients at initial diagnosis. **(A–C)** PBMCs collected from AML patients at initial diagnosis were stimulated *in vitro* with anti-CD3 and anti-CD28 before intracellular staining of IL-2, IFN-γ and TNF-α (n=28). Flow cytometry representative data (left) and summary plots (right) show the expressions of IL-2 **(A)**, IFN-γ **(B)** and TNF-α **(C)** in indicated CD8 subpopulations (CD26^low^PD-1^+^ (fraction I) and CD26^high^PD-1^+^ (fraction III)). **(D, E)** The expression of Granzyme B and perforin in each CD8 subpopulation. Representative data (left) and summary graphs (right) are shown. *P* values were obtained by paired Student t-test or Wilcoxon signed rank test. *P<0.05, **P<0.01, ***P<0.001, ****<0.0001.

To assess the functional status of CD26^low^PD-1^+^ CD8 T cells, we performed an *in vitro* assay to examine intracellular cytokine productions by CD8 T cells upon anti-CD3 and anti-CD28 stimulation. PBMCs from untreated AML patients were used in this study. CD8 T cells were gated by CD26^low^PD-1^+^ vs. CD26^high^PD1^+^ and intracellular production of IFN-γ, IL-2, and TNF-α by each cell subpopulation was assessed by flow cytometry analyses. As shown in **Figures 5A–C**, the CD26^low^PD-1^+^ CD8 T cells had significantly lower production of IFN-γ, IL-2, and TNF-α compared with CD26^high^PD-1^+^ CD8 T cells (IFN-γ:13.51% vs. 21.17%, *P*=0.003; IL-2:2.69% vs. 18.63%, *P*<0.0001; TNF-α: 5.27% vs. 8.05%, *P*=0.0163). We also evaluated the intracellular expression of Granzyme B and perforin in each cell subpopulation as an indication of killing capacity. Interestingly, we found a significantly increased level of Granzyme B and perforin in CD26^low^PD-1^+^ CD8 T cells compared to that in CD26^high^PD-1^+^ CD8 T cells (**Figures 5D, E**). The discrepancy between cytokine production and Granzyme B/perforin expression in the functional status of exhausted T cells has been observed in multiple studies (3, 24–27). It is suspected that terminally exhausted T cells may lose energy to secret Granzyme B/perforin, leading to the intracellular accumulation of these molecules (28, 29). Collectively, our data demonstrate that CD26^low^PD-1^+^ CD8 T cells are functionally impaired demonstrated by reduced cytokine production, consistent with the status of exhaustion. In addition, they displayed higher intracellular expression of Granzyme B/perforin, suggesting a terminal exhausted status.

## Discussion

In this study, we performed comprehensive phenotypic and functional analyses on the T cells of PBMCs collected from AML patients and healthy controls. We focused our study on the impact of CD26 expression on T cells and discovered that CD26^low^PD-1^+^ CD8 T cells were associated with AML progression. Specifically, the percentage of this cell fraction was significantly higher in patients with newly diagnosed AML (high leukemia burden) compared to that in patients who achieved CR (no leukemia) or healthy controls. Our subsequent studies on CD26^low^PD-1^+^ CD8 T cells from newly diagnosed AML patients demonstrated that this cell population carries features of T cell exhaustion, manifested by higher frequency of T_EMRA_ differentiation, increased expression of inhibitory receptors and exhaustion-related transcription factors, and functional defects. To our knowledge, this is the first report to uncover the important role of CD26 expression on CD8 T cells in AML.

Increased expression of PD-1 is an essential marker for T cell exhaustion. Multiple studies including ours have demonstrated an up-regulation of PD-1 on T cells from AML patients who have disease relapse (2, 5, 8, 30–33). However, T cell expression of PD-1 was not increased in AML patients at initial diagnosis (32). It may be attributed to the heterogeneity of PD-1^+^ T cells at this particular disease status, thus PD-1 alone is inadequate to distinguish exhausted T cells from T cells of other functional status. In fact, elegant studies of mouse models of chronic viral infection have shown a higher frequency of PD-1^+^ CD8 T cells at activation phase short after viral infection as well as in the exhaustion status later in the chronic phase (34–36). It is possible that PD-1^+^ T cells in newly diagnosed AML patients are diverse in their functional status. In our current study, we further dissected PD-1^+^ CD8 T cells into three subpopulations based on their expression of CD26. We made novel findings that CD26^low^PD-1^+^ CD8 T cells were phenotypically and functionally consistent with exhaustion status. Importantly, the frequency of CD26^low^PD-1^+^ CD8 T cells was significantly higher in newly diagnosed AML patients compared to that in healthy controls or AML patients in CR. Therefore, two-dimensional analysis testing both CD26 and PD-1 on CD8 T cells provides an optimal strategy to identify exhaustion T cells in newly diagnosed AML. With a hypothesis that higher frequency of exhausted T cells leads to poor prognosis due to compromised anti-leukemia immune response, we performed analyses on the data of newly diagnosed AML patients to determine the impact of CD26^low^PD-1^+^ CD8 T cells on clinical outcome. Twenty-one AML patients whose overall survival (OS) were evaluable (medium follow-up time:1744 days) were divided into two groups based on their CD8 T cells expression level of CD26^low^PD-1^+^. We observed that patients with high percentage of CD26^low^PD-1^+^ CD8 T cells displayed a trend of lower OS compared to that of low percentage subgroup (median: 372 vs. 1369 days; P=0.231; **Supplemental Figure 3**). No statistical significance was achieved likely due to limited sample size. Further studies in larger cohorts of patients are warranted to make a conclusion. If validated, CD26^low^PD-1^+^ CD8 T cells could be a prognostic biomarker for newly diagnosed AML.

TOX has been considered as an essential transcription factor governing terminally exhausted T cells, whereas TCF1 is more functional in progenitor exhausted T cells (37–41). Our finding that CD26^low^PD-1^+^ CD8 T cells expressed higher TOX and lower TCF1 suggests that these cells are more toward terminal exhaustion. Consistently, we observed that CD26^low^PD-1^+^ CD8 T cells contain higher frequency of Eomes^+^T-bet^low^ cells, a pattern that is observed in terminal exhaustion. Furthermore, whereas being functionally impaired, evident by less cytokine production capacity, CD26^low^PD-1^+^ CD8 T cells express more granzyme B and perforin, another feature of terminal exhausted CD8 T cells. Taking together, our data support the notion that CD26^low^PD-1^+^ CD8 T cells in newly diagnosed AML represent a terminally exhausted T cell population. This finding has significant clinical impact. Multiple studies in preclinical models have demonstrated that reversing T cell exhaustion by PD-1 inhibition effectively reduces leukemia burden (6, 9); however, results from clinical studies applying PD-1 blockade to AML patients are largely negative (11–13). An important strategy to improve treatments targeting T cell exhaustion is to define predictive biomarkers to identify patients who are likely to respond to the treatment. It becomes clear that terminally exhausted T cells have minimal response to PD-1 blockade, a main mechanism for resistance to checkpoint inhibitors (25, 35). Our novel finding that CD26^low^PD-1^+^ CD8 T cells are terminally exhausted is compelling: high frequency of this cell population in newly diagnosed AML may lead to poor response to PD-1-targeting agents. Therefore, optimizing clinical trial design by selecting patients with low percentage of CD26^low^PD-1^+^ CD8 T cells has a strong potential to improve efficacy of treatment with PD-1 blockade.

In contrast to CD26^low^PD-1^+^ CD8 T cells, we observed that CD26^high^PD-1^+^ CD8 T cells are highly functional evident by predominant differentiation stage of T_EM_ and potent cytokine release upon *in vitro* stimulation with anti-CD3/CD28. This is in line with the findings of Bailey et al. that CD26^high^ CD4 T cells showed strong anti-tumor activity when transferred into mouse models of solid tumors (22). Given the fact that CD26 has a costimulatory function in activating T cells, sitagliptin, a CD26/DPP4 inhibitor, was applied to patients undergoing allogeneic stem cell transplantation as graft vs. host disease (GVHD) prophylaxis in a phase 2 clinical trial (42). Low incidence of grade II to IV acute GVHD was observed. A major concern is a decrease of graft vs. leukemia (GVL) effect due to CD26 inhibition. Encouragingly, one-year relapse rate was comparable with historical controls, indicating a preservation of GVL. However, longer follow-up and randomized studies are needed to draw a conclusion. It has been reported that CD26 is overexpressed on tumor cells and promotes metastasis of solid tumors (43–46). Several CD26 inhibitors have been tested for their direct cytotoxic effect against tumors in preclinical models (47–49). Although promising results were observed in some studies, most data were inconclusive or negative. It is possible that systemic treatment with CD26 inhibitors significantly suppresses T cell function and compromises the anti-tumor effect. Combining approaches of regaining T cell activity while inhibiting CD26 would be helpful to circumvent this obstacle. Further investigation of the mechanisms by which CD26 mediates T cell response is essential to identify such therapeutic targets.

Of note, we also examined the differentiation status (T_N_, T_CM_, T_EM_ and T_EMRA_) in subsets of CD26^low^PD-1^+^, CD26^int^PD-1^+^ and CD26^high^PD-1^+^ CD8 T cells in healthy donor (HD) and CR groups. We observed that the differentiation status of each subset among HD and CR samples was similar to that in newly diagnosed samples (Dx) (**Supplemental Figures 4A, B**; **Figure 2**). In addition, expression patterns of TIGIT and CD226 in each subset among HD and CR samples was also similar to that in Dx samples (**Supplemental Figures 4C, D**). These observations indicate that each subset (CD26^low^PD-1^+^, CD26^int^PD-1^+^ or CD26^high^PD-1^+^ CD8 T cells) carries a unique feature that is not altered in different clinical settings (HD, Dx, or CR). Instead, the change in frequencies of the subsets reflects the disease specificity, thus we observed significantly increased frequency of CD26^low^PD-1^+^ CD8 T cells in Dx samples, compared to that in HD and CR (**Figure 1**).

It is unclear how CD26^low^PD1^+^ CD8 T cells are increased in AML progression. We examined the apoptosis of CD26^low^PD-1^+^ vs. CD26^high^PD-1^+^ CD8 T cells by evaluating the expression of CD95 and Annexin V. We observed no significant differences between the two subsets (**Supplemental Figures 5A, B**. In addition, when proliferation was assessed by evaluating the expression of Ki-67, no significant difference was observed either (**Supplemental Figure 5C**). Therefore the increased frequency of CD26^low^PD-1^+^ CD8 T cells in AML progression is unlikely due to increased apoptosis of CD26^high^ T cells or altered proliferation in each subset. We further assessed the frequency of CD26^low^PD-1^+^, CD26^int^PD-1^+^ and CD26^high^PD-1^+^ CD8 T cells in the bone marrow of newly diagnosed AML patients, and observed that the percentage of each subset was comparable to peripheral blood (n=5, **Supplemental Figure 6**). So the increase of CD26^low^PD-1^+^ CD8 T cells is unlikely due to migration between blood and bone marrow. As the increase of CD26^low^PD-1^+^ T cells is associated with a decrease in CD26^int^PD-1^+^ T cells (**Figure 1C**), we suspect that differentiation of naïve CD26^int^PD-1^+^ T cells upon AML stimulation may be contributing. Our observation is different from the findings of Bozorgmehr et al, in which apoptosis of CD26^high^ T cells is increased in CLL (50). The discrepancy is liked due to different disease context. This highlights the heterogeneity of T cell responses and importance of disease context-specific studies.

In summary, we made novel observations that CD26^low^PD-1^+^ CD8 T cells are increased in newly diagnosed AML patients, and their phenotypic and functional features are consistent with terminal exhaustion status. These findings suggest a prognostic and predictive value of CD26 in AML, providing pivotal information to optimize the immunotherapy for this devastating cancer.

## Data availability statement

The raw data supporting the conclusions of this article will be made available by the authors, without undue reservation.

## Ethics statement

The studies involving human participants were reviewed and approved by Penn State Hershey Medical Center. The patients/participants provided their written informed consent to participate in this study.

## Author contributions

HuZ and BJ designed the experiments, performed the research, analyzed the results and wrote the manuscript. CA and CZ performed the research and analyzed the results. KM, SM, WE, SM, JC, BW, NS, KR, and MN acquired samples, managed patients and discussed the data. JS discussed the data and reviewed the manuscript. HoZ conceived the concept, designed the experiments, oversaw the interpretation and presentation of the data, and wrote the manuscript. All authors contributed to the article and approved the submitted version.
