# Supplementary material for: CD26lowPD-1+ CD8 T cells are terminally exhausted and associated with leukemia progression in acute myeloid leukemia
Source: Front Immunol. 2023 Jun 29;14:1169144. doi: 10.3389/fimmu.2023.1169144 (PMC10338956; doi:10.3389/fimmu.2023.1169144)
Supplement: Supplementary file 1 [file DataSheet_1.pdf]

Supplementary figure legends

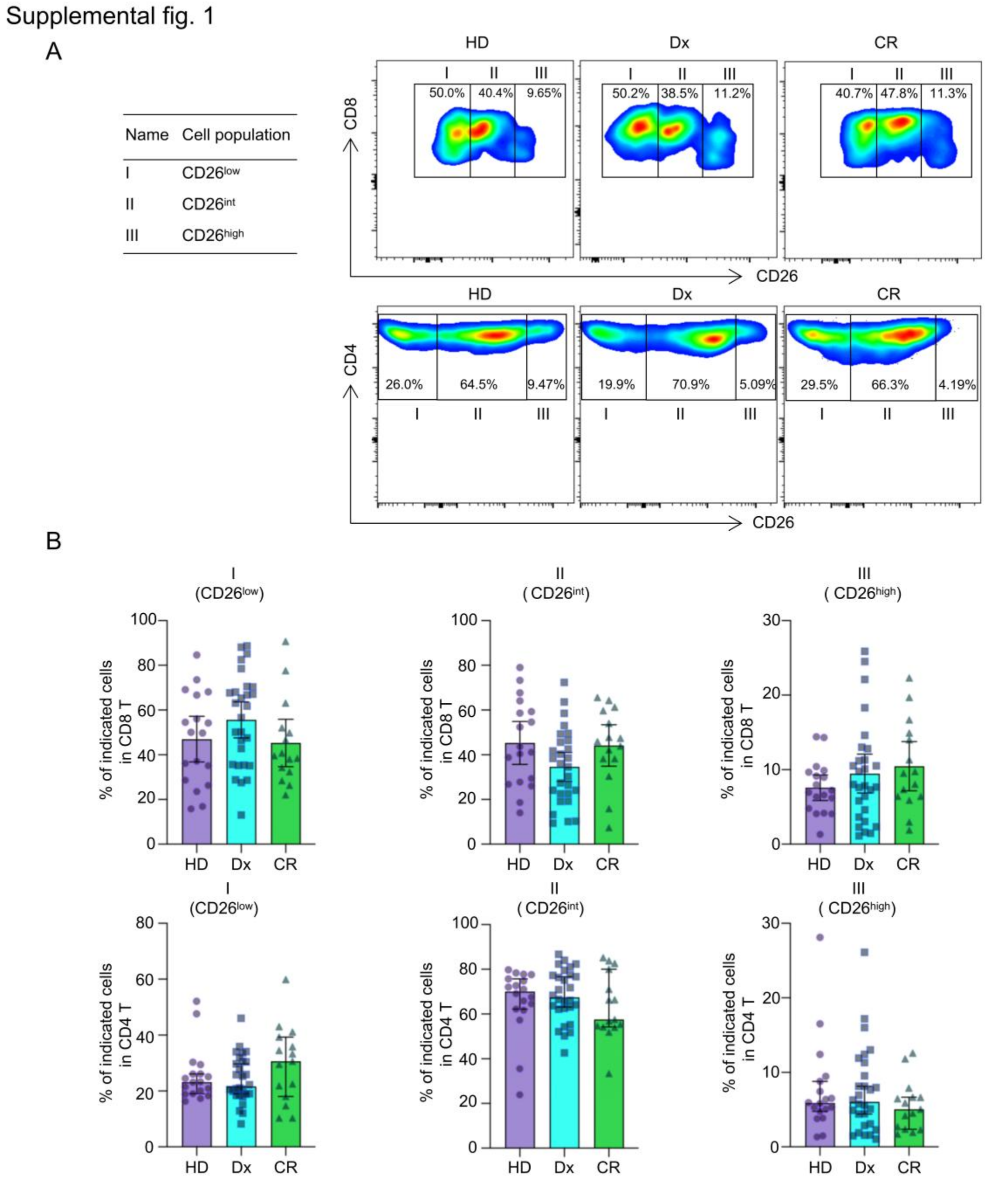

Supplementary figure 1.

The expression of CD26 in CD8 and CD4 T cells in AML patients at initial diagnosis was comparable with that in healthy donors or AML patients in complete remission. PBMCs from healthy donors (HD), AML patients at initial diagnosis (Dx) and AML patients in complete remission (CR) were tested for CD26 expression on CD8 and CD4 T cells by flow cytometry. (A) Based on the levels of CD26 expression, cells were divided into three fractions. The schema of each fraction was shown on the left. Representative flow data (right) from HD, and Dx patients display the percentage of $CD26^{low}$ (fraction I), $CD26^{int}$ (fraction II), $CD26^{high}$ (fraction III) among CD8 and CD4 T cells. (B) Plots of the percentage of each fraction among CD8 and CD4 cells in HD (n=18), Dx (n=28), and AML patients in CR (n=15). Each spot represents data of an individual patient or healthy donor.

Supplemental fig. 2

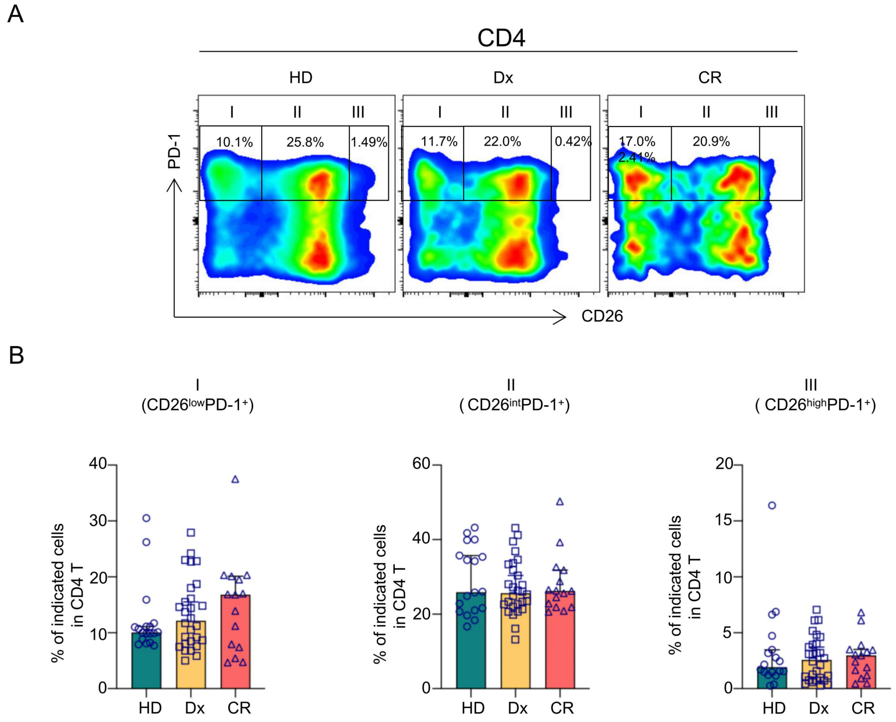

Supplementary figure 2.

The frequency of $CD26^{low}PD\text{-}1^{+}$ CD4 T cells showed no significant difference between healthy donor and AML patients at newly diagnosed and complete remission. PBMCs collected from HD, AML patients at Dx and in CR were assessed for CD26 and PD-1 expression on CD4 T cells by flow cytometry. (A) Representative flow data from HD, Dx, and CR displaying the percentage of $CD26^{low}PD\text{-}1^{+}$(fraction I), $CD26^{int}PD\text{-}1^{+}$ (fraction II), $CD26^{high}PD\text{-}1^{+}$ (fraction III) among CD4 T cells. (B) The summary plots of the frequencies of each fraction among CD4 cells in HD (n=18), Dx (n=28), and CR (n=15). Each spot represents data of an individual patient or healthy donor.

Supplemental fig.3

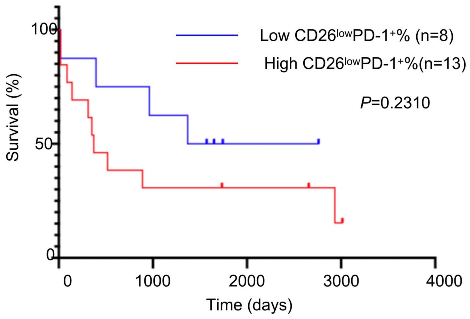

Supplementary figure 3.

The Association of CD26^{low}PD-1^{+} CD8 T cells to the overall survival. Flow cytometry analyses of CD26^{low}PD-1^{+} were performed on PBMCs collected from AML patients at initial diagnosis. The ROC curve was used to predict the reasonable grouping cutoff of low CD26^{low}PD-1^{+} and high CD26^{low}PD-1^{+} in newly diagnosed AML patients. Shown is the overall survival in high CD26^{low}PD-1^{+}% (n=13) vs. low CD26^{low}PD-1^{+}% (n=8) group.

Supplemental fig. 4

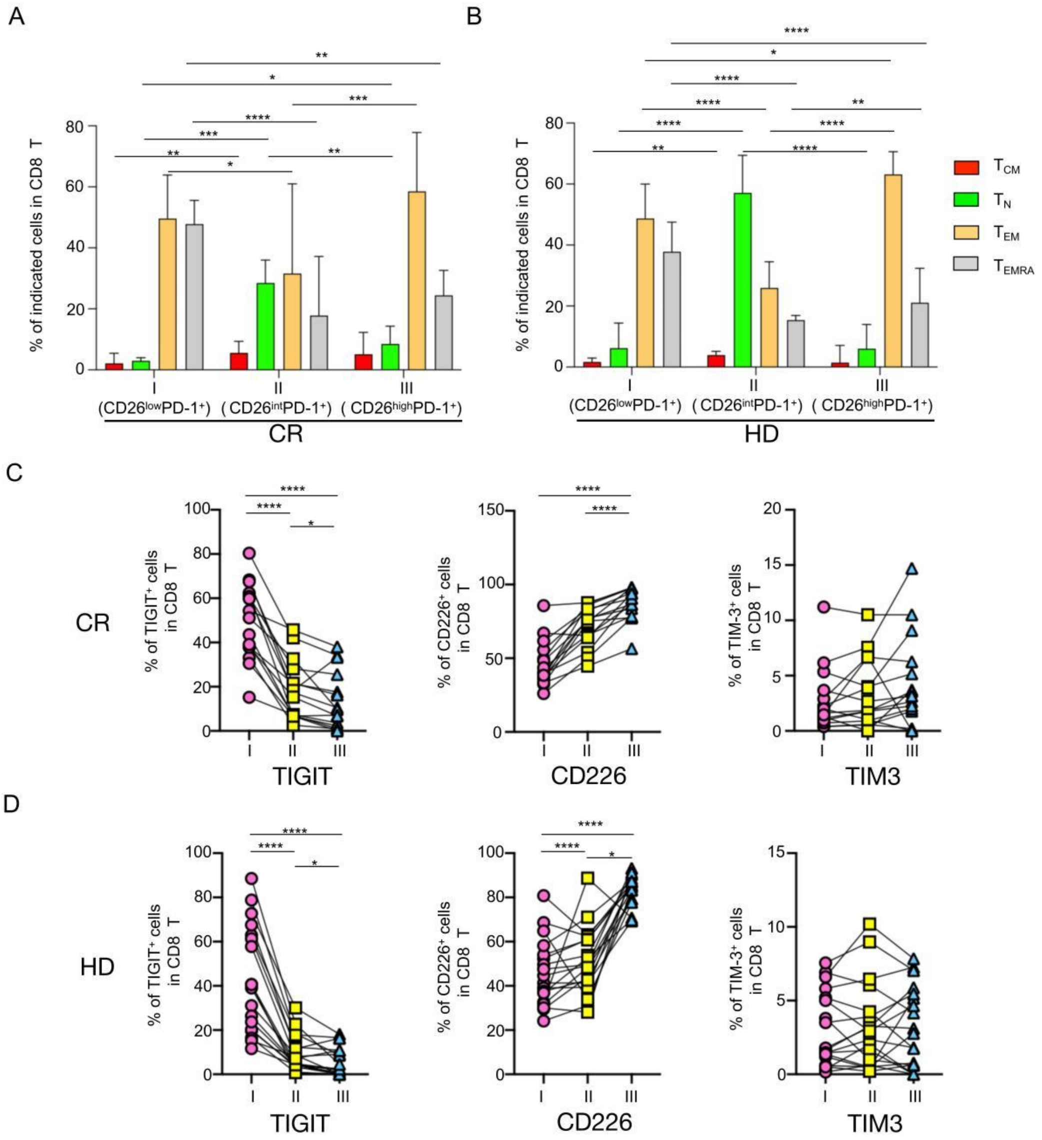

Supplementary figure 4.

The characteristics of the CD8 subsets in HD and CR. Flow cytometry analysis of surface expression of PD-1, CD26, CD45RA, CCR7 was performed on PBMCs collected from HD (n=18) and CR (n=15). (A-B) Summary data for the distribution of Naïve (T_N), central memory (T_{CM}), effector memory (T_{EM}) and terminal differentiated cells (T_{EMRA}) in the three fractions of CD8 T cells ($CD26^{low}PD\text{-}1^{+}$(fraction I), $CD26^{int}PD\text{-}1^{+}$(fraction I) and $CD26^{high}PD\text{-}1^{+}$ (fraction III)) in CR and HD. (C-D) The expression of TIGIT, TIM-3, and CD226 on CD8 T cell subsets in HD and CR was assessed by flow cytometry. Shown are the summary plots. *P* values were obtained by paired Student t-test or Wilcoxon signed rank test. **P*<0.05, ***P*<0.01, ****P*<0.001, *****P*<0.0001.

Supplemental fig.5

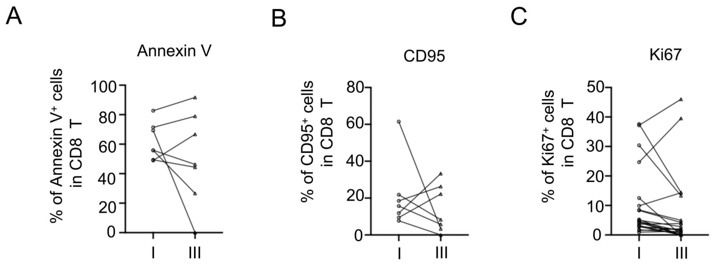

Supplementary figure 5

The apoptosis and proliferation of each CD8 subset in newly diagnosed AML patients. (A-B) The apoptosis was evaluated by the expression of AnnexinV and CD95 in CD26^{low}PD-1^{+}(fraction I) and CD26^{high}PD-1^{+} (fraction III) by flow cytometry. Shown are the summarized plots. (C) The proliferation was assessed by the expression of Ki67 in CD8 fraction I/III. Shown is the summary plot.

Supplemental fig.6

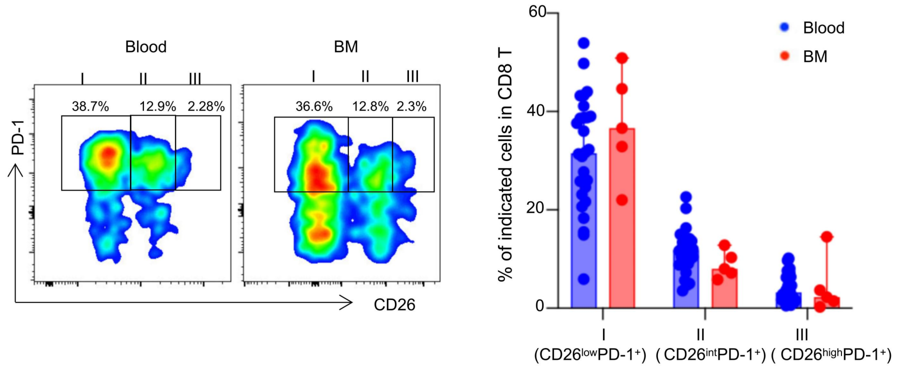

Supplementary figure 6

The frequencies of each CD8 T cell subset in bone marrow (BM) were similar to that in peripheral blood. The representative flow images of CD26^{low}PD-1^{+}(fraction I), CD26^{int}PD-1^{+}(fraction II), and CD26^{high}PD-1^{+} (fraction III) are shown on the right. The summarized plots are on the left.
